# Supplementary figures and images for: Explainable Machine Learning Model for Glaucoma Diagnosis and Its Interpretation
Source: Diagnostics (Basel). 2021 Mar 13;11(3):510. doi: 10.3390/diagnostics11030510 (PMC8001225; doi:10.3390/diagnostics11030510)

# Figure 1. Prediction case 1

Actual : **Healthy**      Predict: **Healthy**

| PSD  | RNFL_S | RNFL_I | RNFL_T | IOP |
|------|--------|--------|--------|-----|
| 1.92 | 142    | 153    | 94     | 13  |

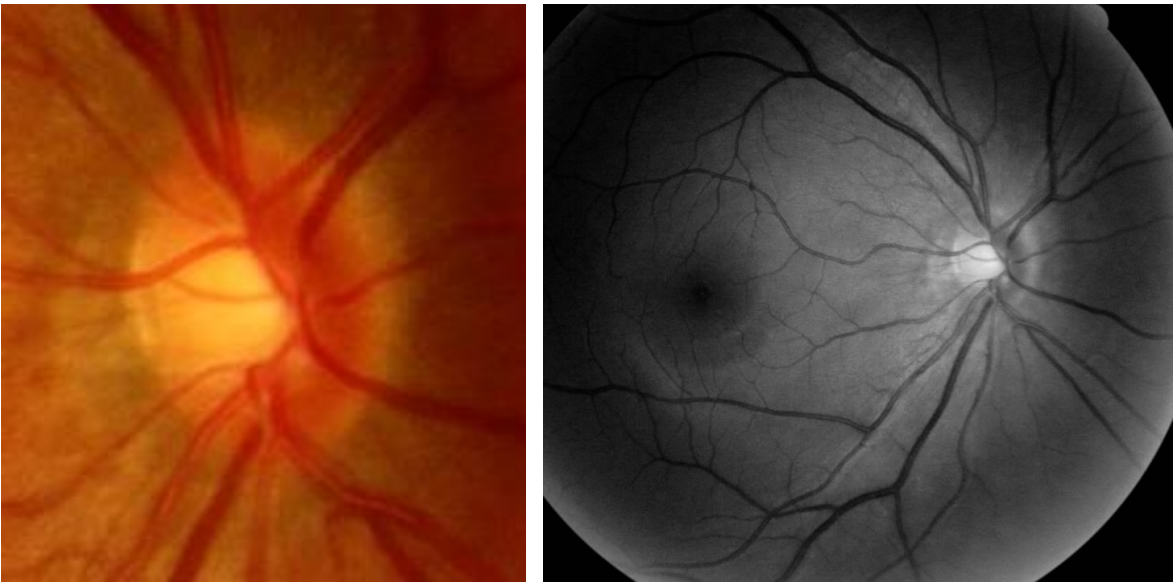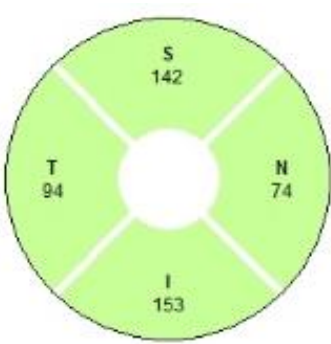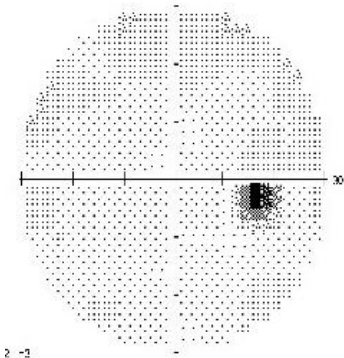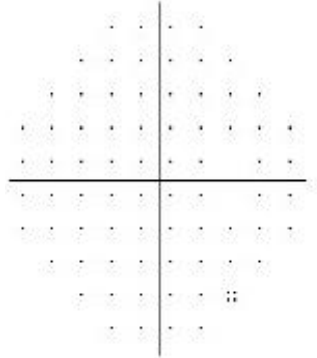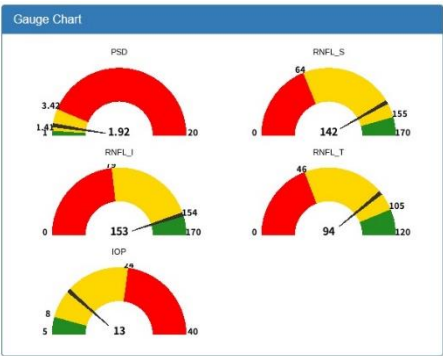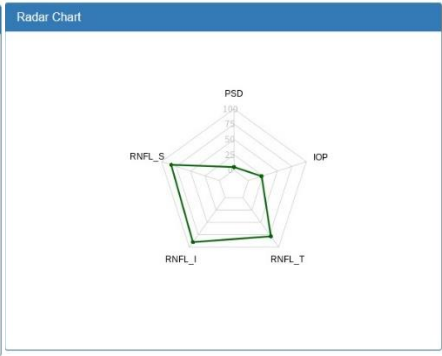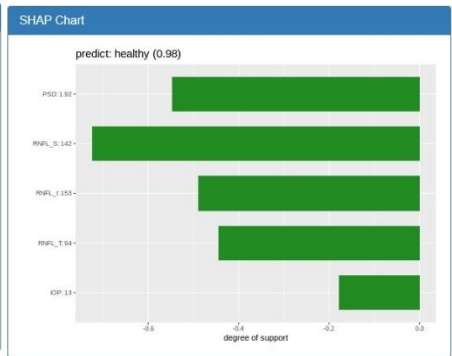

Supplement: Supplementary file 1 [file diagnostics-11-00510-s001.zip › FIG_S1.pdf]

Figure 2. Prediction case 2

Actual : Glaucoma                      Predict: Glaucoma

| PSD   | RNFL_S | RNFL_I | RNFL_T | IOP |
|-------|--------|--------|--------|-----|
| 11.85 | 83     | 41     | 55     | 14  |

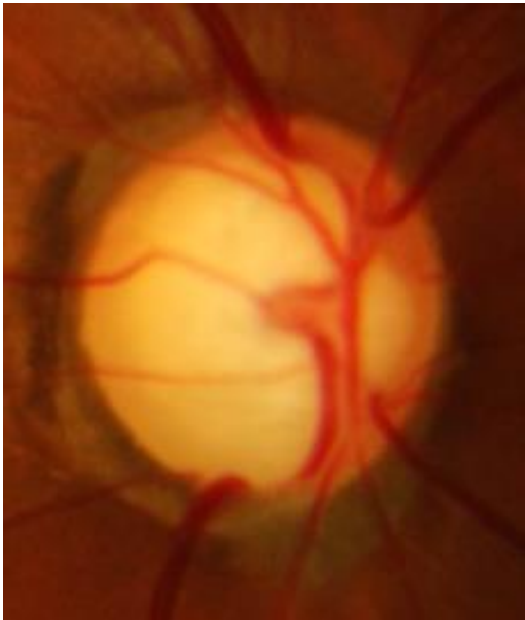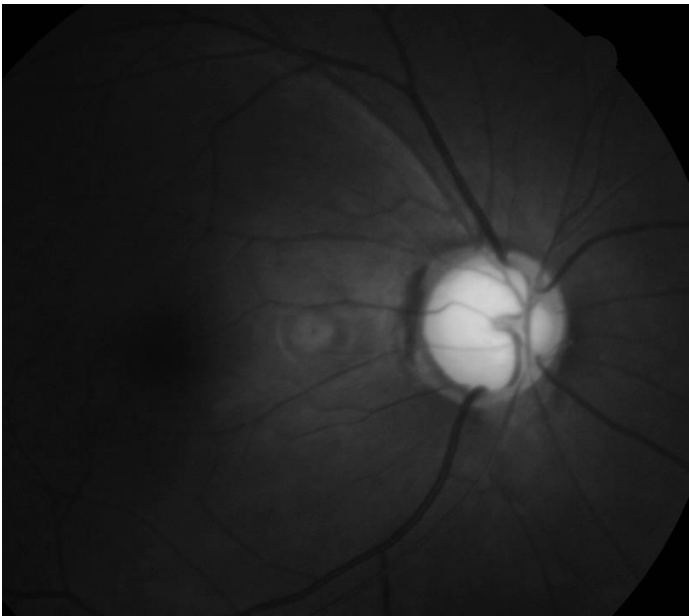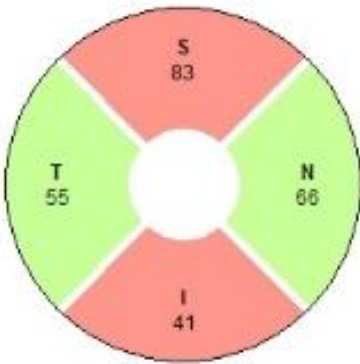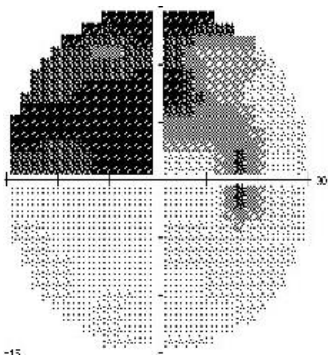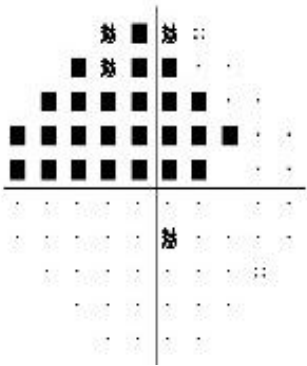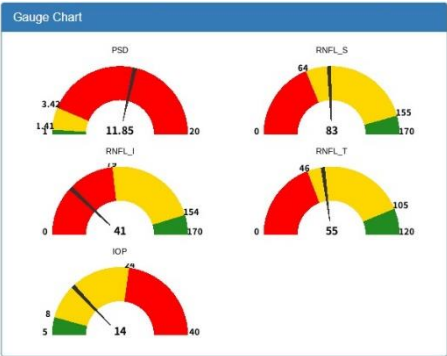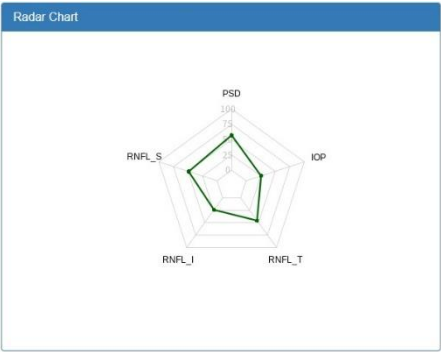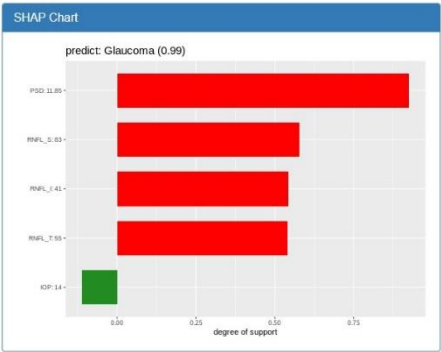

Supplement: Supplementary file 1 [file diagnostics-11-00510-s001.zip › FIG_S2.pdf]

# Figure 3. Prediction case 3

Actual : **Glaucoma**                      Predict: **Healthy**

| PSD  | RNFL_S | RNFL_I | RNFL_T | IOP |
|------|--------|--------|--------|-----|
| 1.53 | 73     | 107    | 71     | 18  |

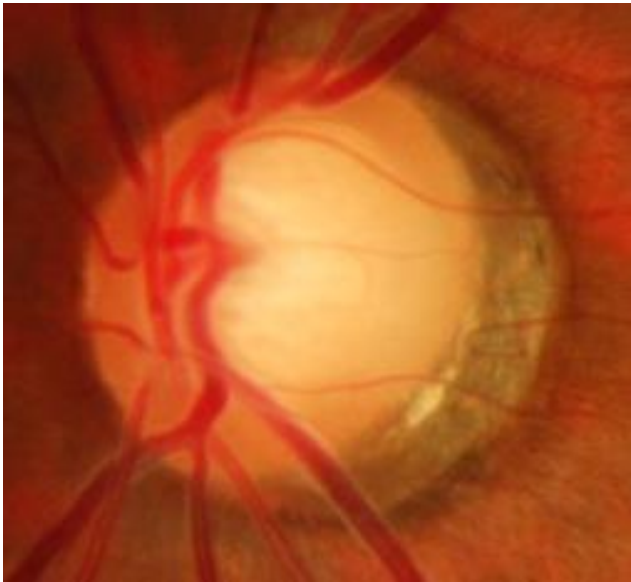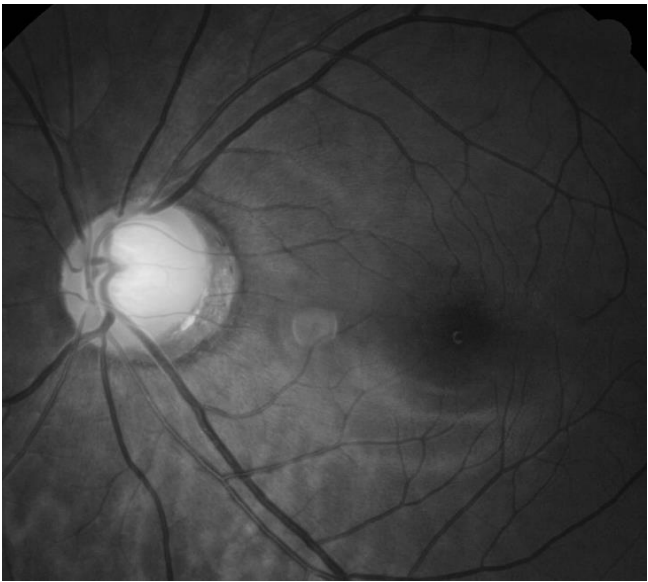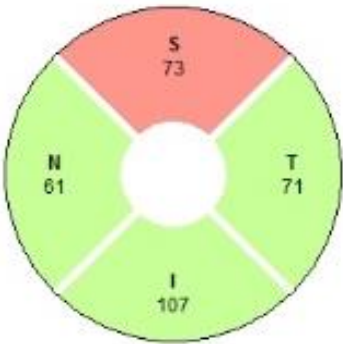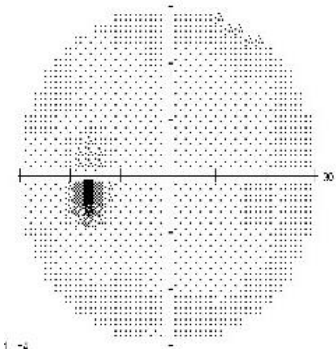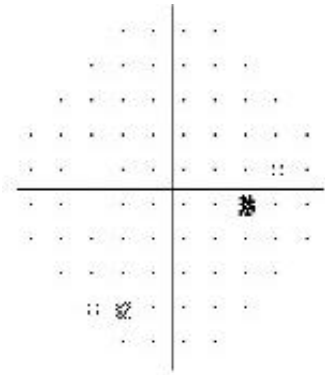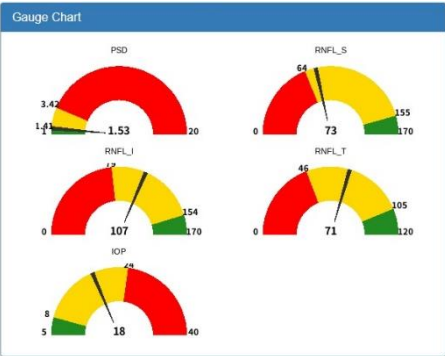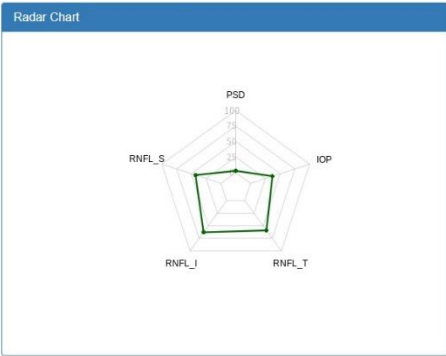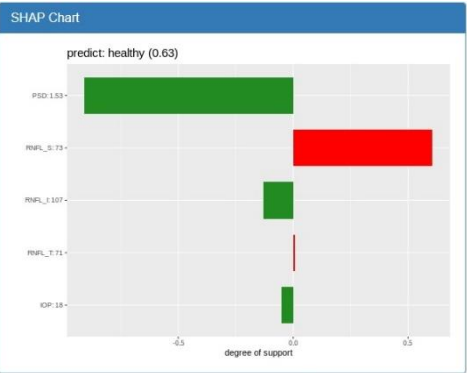

Supplement: Supplementary file 1 [file diagnostics-11-00510-s001.zip › FIG_S3.pdf]

# Figure 4. Prediction case 4

Actual : **Healthy**                      Predict: **Glaucoma**

| PSD  | RNFL_S | RNFL_I | RNFL_T | IOP |
|------|--------|--------|--------|-----|
| 2.76 | 81     | 95     | 73     | 18  |

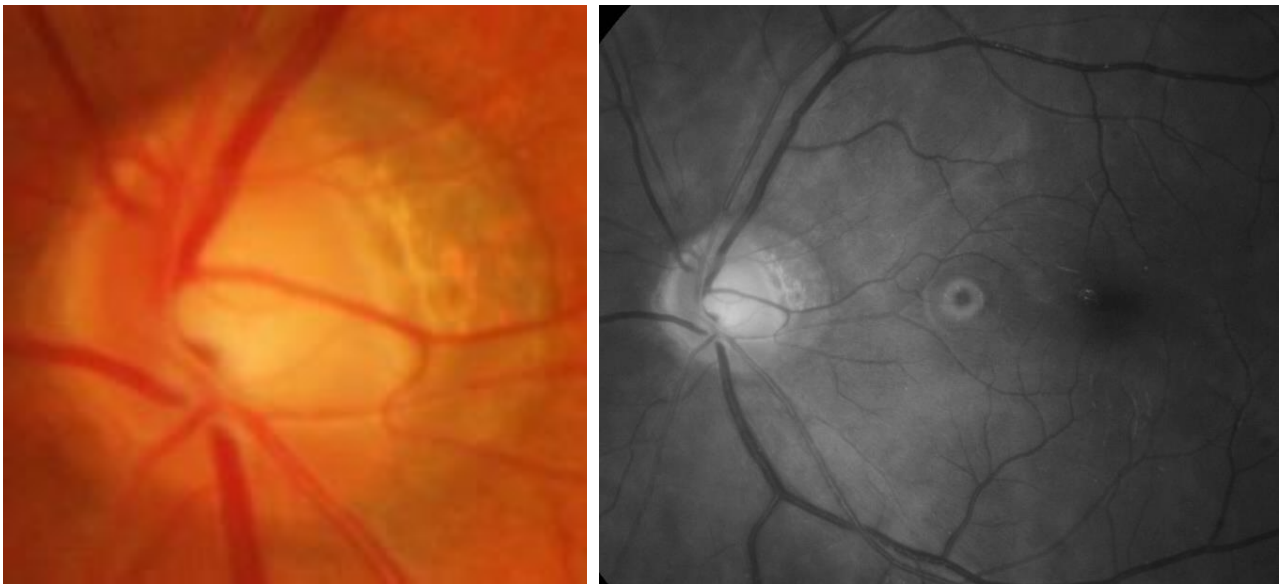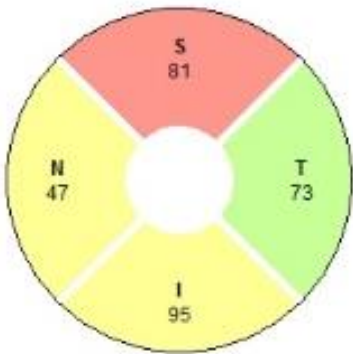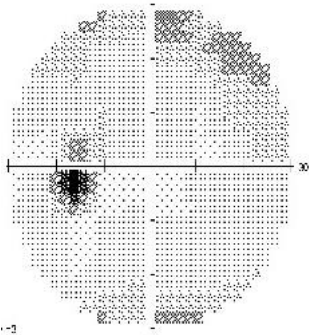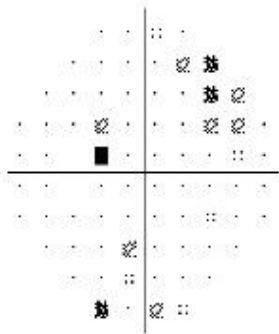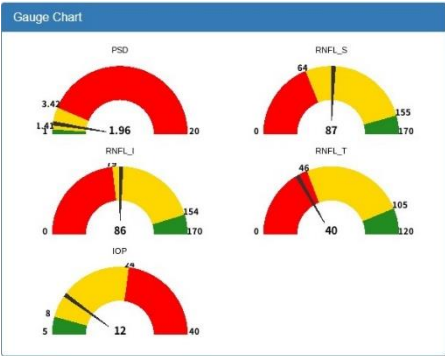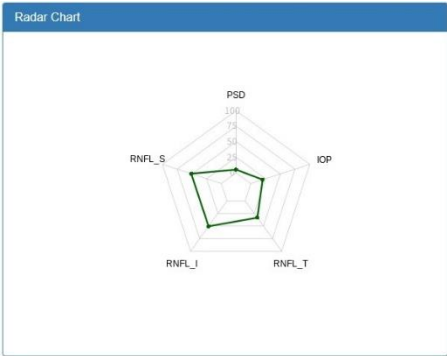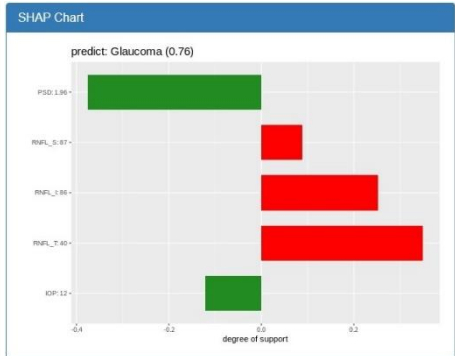

Supplement: Supplementary file 1 [file diagnostics-11-00510-s001.zip › FIG_S4.pdf]

# Figure 5. Prediction case 5

Actual : **Glaucoma**                      Predict: **Healthy**

| PSD  | RNFL_S | RNFL_I | RNFL_T | IOP |
|------|--------|--------|--------|-----|
| 2.31 | 98     | 130    | 60     | 12  |

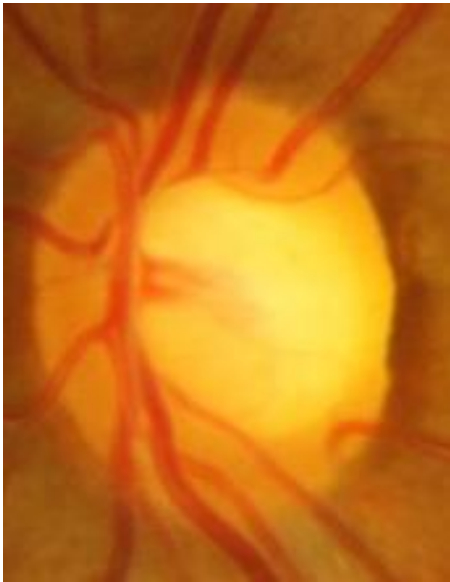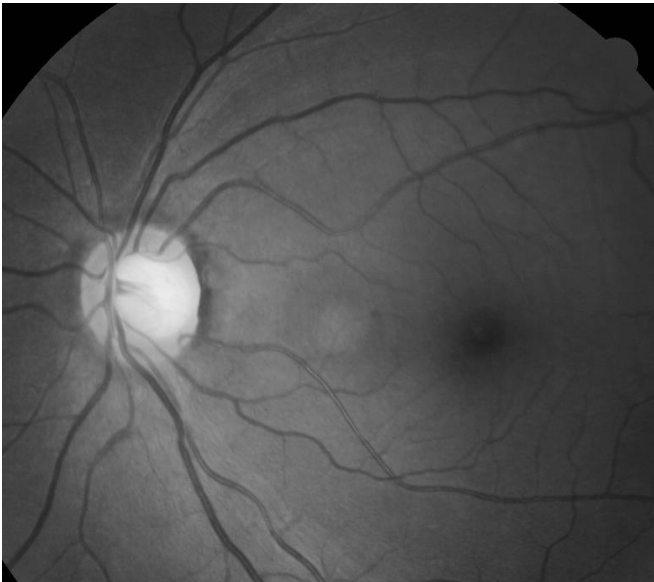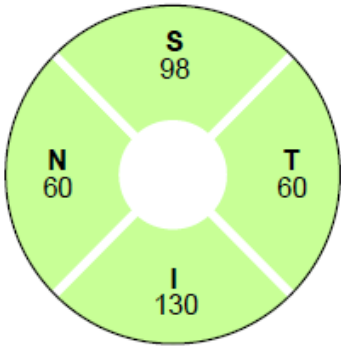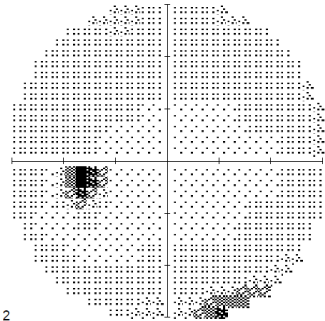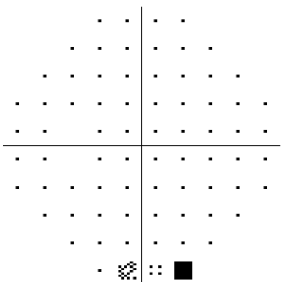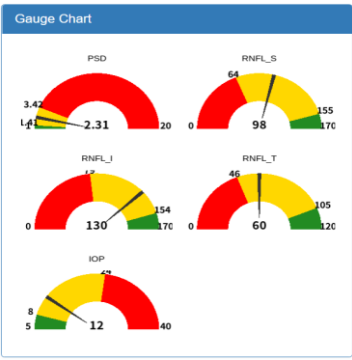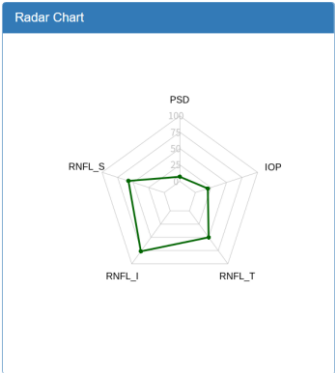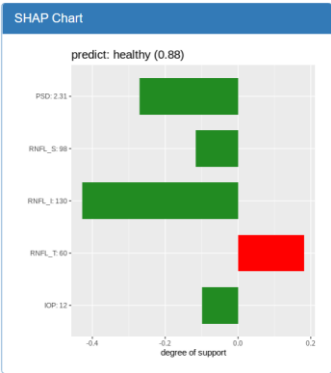

Supplement: Supplementary file 1 [file diagnostics-11-00510-s001.zip › FIG_S5.pdf]

Figure 6. Screen shot of Magellan system

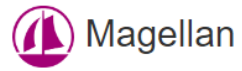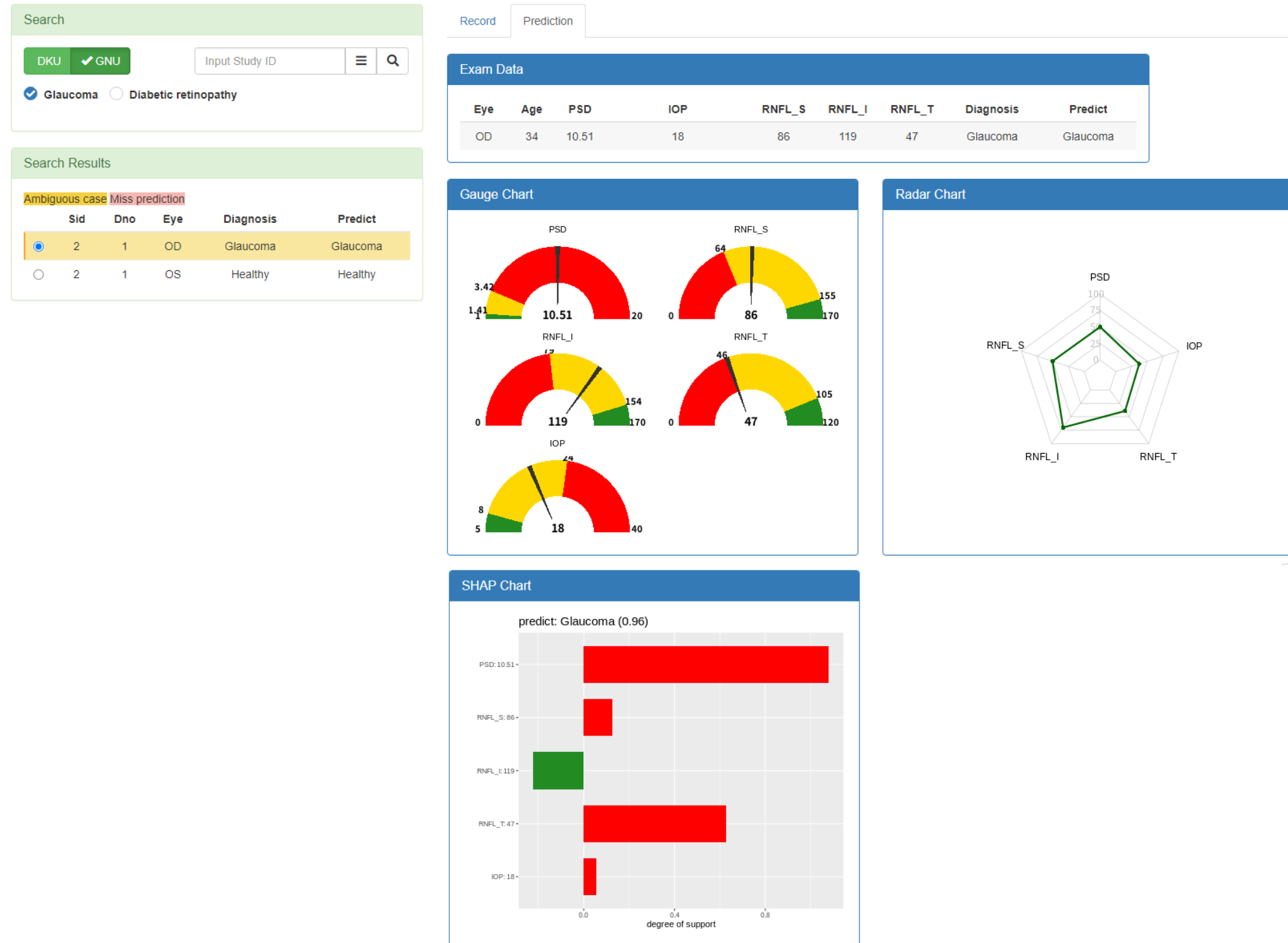

Supplement: Supplementary file 1 [file diagnostics-11-00510-s001.zip › FIG_S6.pdf]
